# Clinically relevant mutations in mycobacterial LepA cause rifampicin-specific phenotypic resistance

**DOI:** 10.1038/s41598-020-65308-2

**Published:** 2020-05-21

**Authors:** Bi-Wei Wang, Jun-Hao Zhu, Babak Javid

**Affiliations:** 1 Centre for Global Health and Infectious Diseases, Collaborative Innovation Centre for the Diagnosis and Treatment of Infectious Diseases, Tsinghua University School of Medicine, Beijing, China; 2 Immunology and Infectious Diseases, Harvard TH Chan School of Public Health, Boston, USA; 3 Beijing Advanced Innovation Center in Structural Biology, Beijing, China

**Keywords:** Bacterial genomics, Antibiotics, Antimicrobials, Microbiology, Bacterial genes

## Abstract

Although all wild-type bacterial populations exhibit antibiotic tolerance, bacterial mutants with higher or lower tolerant subpopulation sizes have been described. We recently showed that in mycobacteria, phenotypically-resistant subpopulations can grow in bulk-lethal concentrations of rifampicin, a first-line anti-tuberculous antibiotic targeting RNA polymerase. Phenotypic resistance was partly mediated by paradoxical upregulation of RNA polymerase in response to rifampicin. However, naturally occurring mutations that increase tolerance via this mechanism had not been previously described. Here, we used transposon insertional mutagenesis and deep sequencing (Tnseq) to investigate rifampicin-specific phenotypic resistance using two different *in vitro* models of rifampicin tolerance in *Mycobacterium smegmatis*. We identify multiple genetic factors that mediate susceptibility to rifampicin. Disruption of one gene*, lepA*, a translation-associated elongation factor, increased rifampicin tolerance in all experimental conditions. Deletion of *lepA* increased the subpopulation size that is able to grow in bulk-lethal rifampicin concentrations via upregulation of basal *rpoB* expression. Moreover, homologous mutations in *lepA* that are found in clinical *Mycobacterium tuberculosis* (Mtb) isolates phenocopy *lepA* deletion to varying degrees. Our study identifies multiple genetic factors associated with rifampicin tolerance in mycobacteria, and may allow correlation of genetic diversity of clinical Mtb isolates with clinically important phenotypes such as treatment regimen duration.

## Introduction

Antibiotic tolerance describes genetically susceptible bacterial subpopulations that are killed more slowly than the bulk population^1,2^. There are a spectrum of phenotypes associated with antibiotic tolerance^3^. The best studied is non-replicating persistence – in which non- or slowly-replicating bacteria are typically multi-drug tolerant^1^. However, increasing evidence, particularly in mycobacteria, suggests that actively replicating bacteria can also be highly drug tolerant^4–9^. We have previously focused on tolerance in actively growing cells to the first-line anti-tuberculous antibiotic rifampicin, which inhibits RNA polymerase (RNAP), and which we termed rifampicin-specific phenotypic resistance (RSPR). We observed that mycobacteria can not only survive, but actively grow in bulk-lethal concentrations of rifampicin. Both specific translational errors involving the indirect tRNA aminoacylation pathway, as well as a paradoxical upregulation of *rpoB* in response to rifampicin mediated RSPR^7,9,10^. Importantly, both mechanisms of RSPR were confirmed in clinical isolates of *Mycobacterium tuberculosis* (Mtb), corroborating the potential clinical relevance of this form of antibiotic tolerance^7,9^. Transposon insertion mutagenesis and deep sequencing (Tnseq) has proven a valuable tool for forward genetics in bacteria. Although it has been used extensively for identification of genetic factors involved in bacterial physiology, host-pathogen interactions, as well as antibiotic resistance^11–15^, investigation of antibiotic tolerance in mycobacteria using Tnseq has been limited^16–18^. Here, we use Tnseq in two models of rifampicin tolerance in *Mycobacterium smegmatis* (Msm) and identify genetic factors implicated in both hypertolerance and hypersusceptibility to rifampicin. In particular, we identify that deletion of the putative translation elongation factor LepA mediates RSPR via perturbation of the physiological transcriptional response of *rpoB*, and show that mutations in *lepA* identified in clinical Mtb isolates phenocopy *lepA* deletion in mediating rifampicin tolerance. Given these mutations are in conserved sites between *M. smegmatis* and *M. tuberculosis*, it is likely their phenotypes will be conserved.

## Results

### Tnseq identifies inactivation of *lepA* as a mediator of rifampicin phenotypic resistance

**Figure 1:**
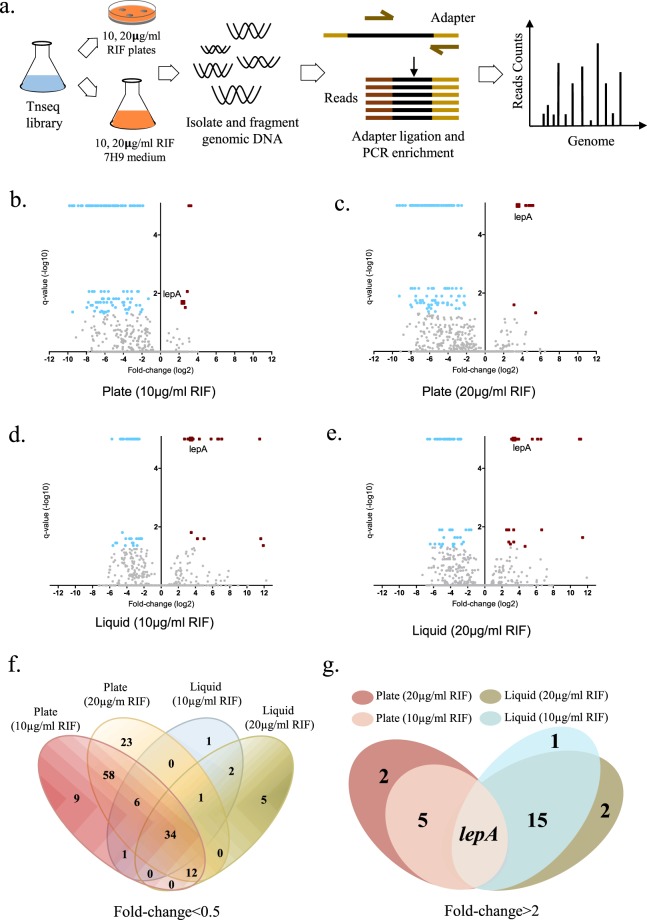
Tnseq identifies genes contributing to rifampicin-specific phenotypic resistance. (**a**) Cartoon illustrating selection strategy. A transposon-insertion mutagenized library of *Mycobacterium smegmatis* (Msm Tn library) was subjected to four different selection conditions as illustrated, following which genomic DNA of survivors was extracted and prepared for transposon insertion site mapping by deep sequencing (see Methods). Volcano plots illustrating significantly enriched Tn reads under selection by rifampicin-agar 10 µg/mL (**b**), or20 µg/mL (**c**), or 7H9-rifampicin 10 µg/mL (**d**) or 20 µg/mL (**e**) after resampling by TRANSIT tool. Genes with insertions enriched log_2_ (<0.5) compared with the input library and q-value < 0.05 are shown with blue dots and those enriched log_2_ (>2) and q-value < 0.05 are shown as red dots. *lepA*(*Msmeg_4556*) is highlighted. See also Datasets 1 and 2. Venn diagrams illustrating overlap in enriched for under-represented (**f**) and over-represented (**g**) Tn insertion reads in the four conditions.

To investigate non-essential genes contributing to rifampicin-specific phenotypic resistance in *Mycobacterium smegmatis*, we first constructed a high density (1.5 × 10^5^ unique clones) transposon-mutagenized library via phage transduction of a *Himar1* transposon that inserts at TA dinucleotides within the genome^12,19^. The library was subjected to 4 different selection conditions: plating on rifampicin-agar at either 10 µg/mL or 20 µg/mL (representing 1x and 2x the plating MIC_90_), or inoculation into 7H9 liquid medium containing 10 µg/mL or 20 µg/mL rifampicin (representing 4x and 8x the liquid culture MIC_90_). We had previously shown that under these conditions, selection resulted in survival of between 3–10% of inoculated bacteria^9^, thus allowing for analysis of survivors by Tnseq. Importantly, survival and growth-mediated tolerance on rifampicin-agar would exclude genetic mutants that induced a non-replicating persister state, allowing us to interrogate alternative mechanisms of tolerance involving actively growing bacteria. Following selection, bacteria were pooled, lysed and genomic DNA extracted, and transposon insertion site frequencies mapped by deep sequencing and compared with the input library prior to selection (Fig. 1a and^12,19^).

Rifampicin resistance is mediated solely by mutations in the rifampicin-resistance determining region of the *rpoB* gene, coding for the essential β subunit of RNAP. Thus, transposon mutagenesis, which targets non-essential genes only, would not select for *bona fide* rifampicin resistant mutants. Therefore our selection strategy would enrich for mutants that were either hyper-susceptible or hyper-tolerant (but not resistant) to rifampicin.

We compared transposon insertion sites that were both under- and over-represented under rifampicin selection by TRANSIT tool^19^ resampling with correction for multiple comparisons (Fig. 1b–e, Fig. S1 and Datasets S1 and S2). In total, transposon insertions in 34 genes were significantly under-represented under all four conditions of rifampicin selection, suggesting that they were conditionally essential for rifampicin tolerance (Fig. 1f and Table S1). By contrast, transposon insertions were over-represented in a single gene, *lepA* (*Msmeg_4556*) under all four conditions (Figs. 1g and S2).

### Mutations in *lepA* identified from clinical isolates confer rifampicin phenotypic resistance

**Figure 2:**
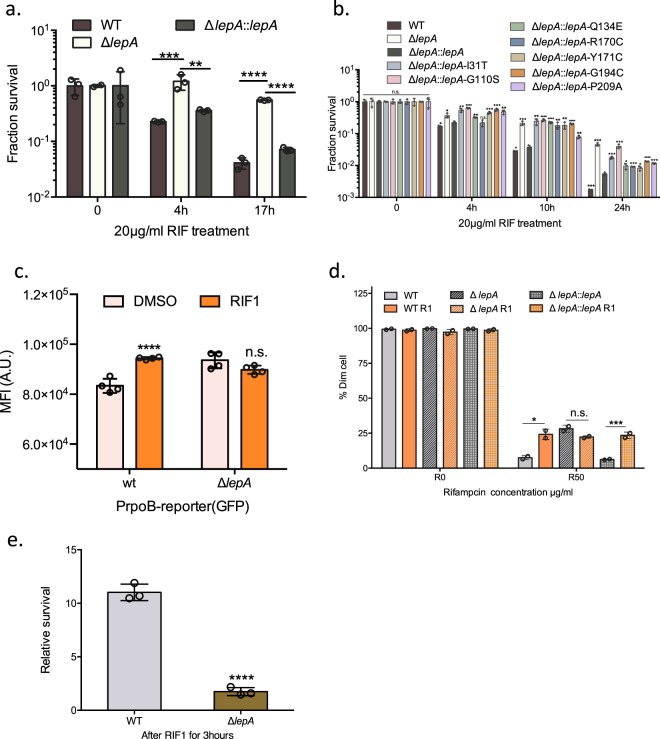
Loss of function in LepA causes rifampicin-specific phenotypic resistance. **(a)** Relative survival of wild-type (WT) *M.smegmatis* (Msm) following treatment with 20µg/mL rifampicin in axenic culture compared with the *lepA* deletion strain (*∆lepA)* and the deletion strain complemented with wild-type *lepA*(*∆lepA::lepA*). **(b)** Relative survival in rifampicin of WT*M. smegmatis* compared with the *lepA* deletion strain (*∆lepA*), or the deletion strain complemented with wild-type *lepA* (*∆lepA::lepA*) or specified mutations in *lepA*. Statistical comparison of means with *∆lepA::lepA* by Student’s t-test is shown. **(c)** Relative green fluorescence, representing expression from the *rpoB-rpoC*-promoter driven GFP of WT Msm and *∆lep A*strains. **(d)** WT or *∆lepA* strains were treated with 1µg/mL rifampicin or carrier for 3hours, following which the bacteria were stained with AF488 and then treated with rifampicin at indicated concentrations or carrier (DMSO) overnight, and proportion of dim cells, representing growing bacteria plotted. **(e)** WT or *∆lepA* strains were treated with 1 µg/mL rifampicin or carrier for 3hours, following which bacteria were pelleted, washed and replated on rifampicin-agar 10µg/mL or non-selective medium. Relative survival of RIF 1-treated compared with untreated bacteria are shown. *p<0.05, **p<0.01, ***p<0.001 and ****p<0.0001 by Student’s t-test. n.s. no significant difference.

Since transposon insertion in *lepA* was identified as a cause for increased rifampicin tolerance in all four experimental conditions, we decided to focus on deletion of *lepA* for further characterization. We constructed a strain of *M. smegmatis* in which the gene coding for *lepA* was deleted by recombineering^7^, *∆lepA*. The minimum inhibitory concentration (MIC) to several anti-mycobacterial antibiotics was similar between wild-type *M. smegmatis* and *∆lepA*, confirming that deletion of *lepA* did not confer altered resistance, including to rifampicin (Table S2). However, the strain lacking *lepA* had significantly greater survival to rifampicin compared with the wild-type parent strain and this phenotype was complemented with wild-type *lepA* (Fig. 2a). There was no increased tolerance to the antibiotics isoniazid or streptomycin (Fig. S3), suggesting that *lepA* deletion was not a cause of non-specific antibiotic tolerance. Of note, a substantial number of clinical isolates of *M. tuberculosis* harbor mutations in the *lepA* gene. We selected a number of mutations identified from clinical isolates (https://platform.reseqtb.org), mapping to conserved residues of the annotated GTP-binding domain of the protein (Dataset S1 and Fig. S4). We then complemented the *∆lepA* strain with either the wild-type or mutated *lepA* genes. Intriguingly, all of the conserved mutations failed to fully complement *∆lepA*, but to variable extents (Fig. 2b), suggesting a degree of loss of function in LepA in a proportion of circulating clinical *M. tuberculosis* isolates.

### LepA deletion increases basal expression of RpoB and blunts rifampicin-induced RSPR

We had previously shown that rifampicin and other inhibitors of RNAP triggered upregulation of *rpoB-rpoC* expression due to the operon-specific promoter architecture. This paradoxical upregulation in *rpoB-rpoC* expression was stringently associated with growing tolerant mycobacteria^9^. We wondered whether deletion of *lepA* was associated with an aberrant response to this tolerance-associated transcriptional response. Using a previously characterized fluorescent reporter, P_(rpoB-rpoC)_-mEmerald, which measures the transcriptional response from the *rpoB-rpoC* promoter, we observed that deletion of *lepA* resulted in a higher ‘basal’ degree of expression from the promoter, but addition of subinhibitory concentrations of rifampicin failed to further upregulate expression (Fig. 2c). We had previously demonstrated that the mycobacterial *rpoB-rpoC* promoter contains two conserved promoters. Expression from the 5′ promoter I dampened the maximal response from the stronger promoter II^9^. Deletion of promoter I or its inhibition by subinhibitory concentrations of rifampicin relieved inhibition of promoter II and caused upregulation of *rpoB-rpoC* expression and hence increased rifampicin tolerance^9^. The *lepA* deletion strain behaved similarly to a strain in which promoter I had been deleted. We therefore hypothesized that deletion of *lepA* would dampen rifampicin-induced rifampicin-specific phenotypic resistance. We used two complementary assays for grower-mediated rifampicin tolerance^9^ to test this hypothesis. In the first assay, the mycobacterial cell wall is fluorescently labelled by a fluorophore (Alexafluor-488), and cells are grown in the presence of bactericidal concentrations of drug. Cells that are nonetheless able to survive and grow in bulk-lethal concentrations of drug will lose fluorescence due to fluorescence-dilution, allowing measurement of the ‘grower’ population^9^. As previously demonstrated^9^, in wild-type *M. smegmatis*, exposure to sublethal concentrations of rifampicin prior to exposure to bulk-lethal concentrations of rifampicin led to significantly increased RSPR. However, although the *lepA*-deletion strain had higher basal rifampicin tolerance, this did not increase upon pre-exposure to sublethal rifampicin (Fig. 2d), and this phenotype was complementable. We also verified the phenotype in the ‘plate-growth’ assay used in the initial screening conditions. Exposure of wild-type *M. smegmatis* to sublethal rifampicin prior to plating on rifampicin-agar (10 µg/ml) led to 10-fold increase in RSPR, with a much more blunted response in a strain deleted for *lepA* (Fig. 2e). Together, these assays demonstrated that *lepA* deletion phenocopied *rpoB-C* promoter I deletion, resulting in increased basal rifampicin tolerance, but without a subsequent increase due to rifampicin exposure.

## Discussion

Our study leverages the power of forward genetics using transposon site insertion and deep sequencing (Tnseq) to identify non-essential mycobacterial genes that cause rifampicin phenotypic resistance. Our screen identified a number of genes previously associated with antibiotic tolerance in mycobacteria, as well as new hits. A number of our hits are in proteins embedded in the cell wall/outer mycobacterial layer and/or implicated in cell-wall integrity, such as MmpL11, Antigen85A, PstS, M_SMEG5782c and LytR. Of these, several have been previously identified as associated with either non-replicating persistence and/or biofilm formation^20–23^. Cellular processes that disrupt cell wall integrity and therefore increase intra-cellular antibiotic concentrations would be expected to increase antibiotic susceptibility across many different antibiotics, and this has been verified for the phosphate transporter Pst^24^. The serine/threonine protein kinase, PknG had also been previously identified as important for mycobacterial adaptation to acid stress and persistence^25,26^. In addition to hits implicated in cell wall integrity and/or environmental sensing, our screen identified a number of genes involved in carbon metabolism, for example ArgG, but in particular for branched-chain amino acid synthesis such as IlvB, IlvE and MetH (Table S1). Although it is conceivable that inhibition of central carbon metabolism and/or branched chain amino acid synthesis might result in a state of non-replicating persistence and multi-drug tolerance, these hits were also identified under the rifampicin-agar plate growth conditions that would have specifically excluded persisters. Therefore the exact mechanism by which disruption of these genes increases rifampicin tolerance without necessarily compromising growth in antibiotics is not known.

We chose to focus on LepA in our study, since that was the only gene in which transposon disruption increased rifampicin tolerance instead of increasing susceptibility under all four experimental conditions. LepA is a highly conserved GTPase, with sequence similarity to EF-G, a translation-associated elongation factor, but the precise cellular function of LepA remains controversial^27–29^. LepA was also identified in another forward genetic screen that specifically investigated intra-cellular concentrations of the fluorescent dye calcein, which was in turn associated with rifampicin tolerance^14^. A recent preprint implicates LepA in the synthesis of a mycobacterial porin, and in turn rifampicin and vancomycin tolerance^30^. However, both our data and that of Rubin *et al*. suggests that *lepA* deletion has potentially wider-ranging perturbations of transcriptional and translational responses. Specifically, our data identifying that the *lepA* deletion mutant has a blunted response to sublethal rifampicin exposure, which phenocopies *rpoB-rpoC* promoter I deletion^9^ suggests that the rifampicin tolerance phenotype may involve multiple, non-mutually exclusive mechanisms.

A potential drawback of transposon mutagenesis screens is that hits only in non-essential genes can be identified. However, loss of function mutations in clinical strains are more likely to occur in these non-essential genes, and therefore identification of clinically-relevant phenotypes associated with such loss of function mutations^31,32^ may allow for patient-specific therapeutic regimens.

## Materials and Methods

### Bacterial culture

*Mycobacterium smegmatis* mc^2^-155 (ATCC) was grown in liquid Middlebrook 7H9 broth (BD) containing 0.5%glycerol, 0.05%Tween 80, 10% (ADS, albumin-dextrose-salt) or on LB (BD) agar at 37 °C. *Escherichia coli* was cultured in LB broth Miller (BD) or on LB agar plates at 37 °C. Where needed, antibiotics were used at the following concentrations: kanamycin (25 μg/ml for *M. smegmatis* and 50 μg/ml for *E. coli*), zeocin (25 μg/ml for *M. smegmatis* and 50 μg/ml for *E. coli*), hygromycin (75 μg/ml for *M. smegmatis* and 150 μg/ml for *E. coli*). Rifampicin (TCI) was dissolved in DMSO (stock concentration as 30 mg/ml) and added to culture medium at indicated concentrations.

### Strain construction

All primers used in this study are listed in Table S3. The ∆*lepA* strain was constructed using Rec-ET homologous recombination system as previously reported^7^. Briefly, a 515 bp DNA fragment upstream of *lepA* (*MSMEG_4556*) was PCR amplified using primers MSMEG_4556_KO_1 and MSMEG_4556_KO_2. Similarly, a downstream 507 bp fragment was amplified using MSMEG_4556_KO_3 and MSMEG_4556_KO_4. A Zeocin resistant marker flanked by two LoxP sites was amplified from a template plasmid pKM_Zeo_lox (a kind gift from Dr. Eric Rubin) using Zeo_F and Zeo_R. The three PCR products were stitched together through PCR with primers MSMEG_4556_KO_1 and MSMEG_4556_KO_4. Fresh Rec-ET expressing competent cells were prepared as previously described, transformed with 2 micrograms of purified stitch-PCR product, and selected on LB agar with 25 μg/ml Zeocin. The L5 site integrating plasmid pML1342 system^33^ was employed to construct complementation strains. Wild-type *lepA* was amplified with primers HindIII_lepA_F and XbaI_lepA_R from *M. smegmatis* genomic DNA by KOD DNA Polymerase (NEB). The *lepA* fragment and pML1342 were digested with restriction endonucleases XbaI and HindIII (NEB). After gel purification using V-RLUTE Gel Mini Purification Kit (ZOMANBIO) fragments were ligated with T4 DNA Ligase (NEB) to obtain plasmid pML_1342_lepA. Plasmids pML_1342_SNPlepA1-7 were constructed with forward primers SNP170_F - SNP682_F and universal reverse primer SNP170_R - SNP682_R using pML_1342_lepA as template. Plasmids pML_1342_SNPlepA1-7 were transformed into *E.coli* DH5α competent cells (CW Biotech) using 150 μg/ml hygromycin for selection. Recombinant plasmids pML1342 containing wildtype *lepA* and mutated *lepA* were transformed into fresh ∆*lepA* competent cells by electroporation equipment (Bio-Rad) with voltage 2500 V, capacitance 25μF, resistance 400Ω. *M. smegmatis* was selected on LB agar plates containing 40 μg/ml hygromycin – see Fig. S5 for PCR validation. The wild-type *lepA* or individual mutated *lepA* genes were integrated into the *M. smegmatis* genome at mycobateriophage L5 attachment site attB. The success of strain construction was confirmed by sequenced results of *lepA* PCR products using mutant strains as templates.

### Transposon mutagenesis library construction

The *M. smegmatis* transposon mutant library was constructed according to reference^12^ with minor changes. Briefly, 100 ml exponentially growing (OD_600nm_ = 0.6–0.8) *M. smegmatis* culture was washed three times with MP buffer (50 mM Tris HCl, pH 7.5, 150 mM NaCl, 10 mM Mg_2_SO_4_, 2 mM CaCl_2_) to remove excess culture medium, and re-suspended in 15 ml MP buffer mixed with freshly prepared phage (2 × 10^11^ plaque forming units). The transduction mixture was incubated at 37 °C for three hours with gentle agitation. 3 ml aliquots were then plated onto each of the 5 LB agar plates (prepared in 25 × 25 cm square petri dishes) containing 20 μg/ml Kanamycin and 0.05% Tween 80. After 3 days of incubation at 37 °C, colonies were scraped off the plates and mixed in 20 mL 7H9 broth with 15% glycerol. 2 ml of the mixture (input library) were immediately subjected to gDNA extraction, while the remaining was kept at −80 °C as 1 ml aliquots.

### Rifampicin selection

Two of the 1 ml frozen transposon library stocks were thawed on ice, and recovered in 50 ml fresh 7H9 medium for 3 hours at 37 °C. For agar-plate based selection, aliquots of the recovered library containing 5 × 10^4^ or 2 × 10^5^ bacteria were plated on each of 20 agar plates (prepared in 15 cm × 15 cm round petri dishes) supplemented with 10 μg/ml or 20 μg/ml rifampicin, respectively. For selection in liquid culture, 10^9^ bacteria from the recovered library were pelleted and inoculated into flasks containing 100 ml fresh 7H9 medium and 10 μg/ml or 20 μg/ml rifampicin. All agar plates were covered in foil and kept at 37 °C. After 5 days (1 more day after colonies became visible), colonies from each selection condition were scraped off the plates, washed once with fresh 7H9 medium and once with TE buffer (10 mM Tris-HCl, 1 mM EDTA at pH 9), then re-suspended in 2 ml TE buffer. The two liquid cultures were kept at 37 °C with constant shaking (200 rpm) for 36 hours, washed twice with TE buffer, then re-suspended in 2 ml TE buffer. The four samples were stored at −80 °C for further genomic DNA extraction.

### Transposon sequencing library preparation

Genomic DNA extraction and sequencing library construction were conducted as described in reference^34^. DNA concentrations of the PCR-amplified pre-sequencing samples were quantified using Qubit 2.0 (dsDNA, High-sensitivity kit, Invitrogen) and adjusted to the same concentration using nuclease-free water. The libraries were then pooled and subjected to high-throughput sequencing with the Miseq platform according to manufacturer’s instructions.

### Sequencing data analysis

Raw data was downloaded from MiSeq local server and decompressed before further analysis. TPP pipeline from TRANSIT^19^ was used for pre-processing of raw data, and processed reads were mapped to the *M. smegmatis* mc^2^-155 genome (GenBank accession number GCA_000015005.1) using BWA (Burroughs-Wheeler Aligner). Read1 and Read2 were used to match the terminus of the Himar1 transposon and to extract random barcodes respectively. Unique “template” counts at each TA sites were generated as wig formula. Resampling test module from TRANSIT was used in testing gene conditional fitness cost under each selection condition, which is a classical permutation test in statistics. TTR (trimmed total reads) was used as the normalization method in resampling. Significance of differences between each conditional selection and non-selection was represented by log2-fold change through comparison with a resampling distribution that is generated from randomly reshuffling of the observed counts at TA sites in the region among all datasets. Relative P-value were generated within 10^4^ permutations according to the proportion of reshuffling samples that have more significant difference than in the actual experimental data.

### RSPR killing curve assay

Wild type *M. smegmatis* or its derivatives were cultured in 7H9 liquid medium until exponential growth phase i.e. optical density (OD_600nm_) reached 0.6–0.8. The cultures were then diluted in 7H9 medium to final OD_600nm_ = 0.05, and supplemented with rifampicin at indicated concentrations. Samples from each treatment were taken at 0 hour, 4 hours and 17 hours after inoculation, washed and subjected to several 10× dilutions, then plated on antibiotic-free LB plates. Fractional survival was estimated as previously described^9^.

### Rif-induced RSPR plating assay

Wild-type *M. smegmatis*, or its derivatives were cultured in 7H9 liquid medium until optical density (OD_600nm_) reached 0.6–0.8. 1 ml bacteria was inoculated in 7H9 medium with 1 μg/ml rifampicin or carrier (DMSO). After 3 hours treatment, bacteria were collected and washed with PBST (PBS containing 0.05% tween80) and several 10× dilutions were plated on antibiotic-free or rifampicin agar plates as indicated. RSPR was calculated by the ratio of CFU between plates with and without rifampicin. Relative survival represents the fold-change of rifampicin tolerance with 1 μg/ml rifampicin pre-treatment.

### Alexa-fluor-488 (AF488) assay

The AF488 assay was adapted from reference^9^ with some modifications. SNAP-Surface Alexa Fluor 488 (NEB) was dissolved in DMSO for 5 mg/ml stock solution and stored at −80 °C for up to several weeks. 2 ml of the strain to be tested was cultured in 7H9 medium until exponential growth phase with optical density (OD_600nm_ = 0.6–0.8). 1 ml bacteria were inoculated in 7H9 medium with and without 1 μg/ml rifampicin respectively for 3 hours. After two washes with1ml PBST, bacteria was re-suspended in 50 μl diluted AF488 (working concentration 200 μg/ml). After incubating in the dark at room temperature for 5 min, bacteria were transferred into a fresh tube and then washed twice more with 1 ml PBST. Bacterial pellets were re-suspended in 500 μl 7H9 fresh medium. 100 μl of the suspension were inoculated into 7H9 medium containing 0 μg/ml, 10 μg/ml, 50 μg/ml rifampicin. After 16 hours culture in 37 °C shaking incubator in the dark, 100ul samples were fixed by 100ul 4% Paraformaldehyde (PFA) at room temperature for 20 min. BD Accuri C6 desktop flow cytometry was used to collect fluorescence intensity with 488 nm excitation laser and 533 nm emission filter. % Dim cells representing the relative percentage of growing cells were analyzed by Flow-Jo software.

### Statistical tests

All experiments were repeated in at least 3 times independently. Data are shown as means ± SD. Differences in means were calculated using Student’s t-test.

## Supplementary information

Supplementary Information.

Supplementary Information2.

Supplementary Information3.

Supplementary Information4.

Supplementary Information5.
