# Supplementary material for: Clinically relevant mutations in mycobacterial LepA cause rifampicin-specific phenotypic resistance
Source: Sci Rep. 2020 May 21;10:8402. doi: 10.1038/s41598-020-65308-2 (PMC7242378; doi:10.1038/s41598-020-65308-2)
Supplement: Supplementary file 5 — Supplementary Information5. [file 41598_2020_65308_MOESM5_ESM.pdf]

Title: Clinically relevant mutations in mycobacterial LepA cause rifampicin-specific phenotypic resistance

Authors: Bi-Wei Wang[a], Jun-Hao Zhu[a,b] and Babak Javid[a,c#]

Affiliations:

[a] Centre for Global Health and Infectious Diseases, Collaborative Innovation Centre for the Diagnosis and Treatment of Infectious Diseases, Tsinghua University School of Medicine, Beijing, China

[b] Immunology and Infectious Diseases, Harvard TH Chan School of Public Health, Boston, USA

[c] Beijing Advanced Innovation Center in Structural Biology

Supplementary Figures

a.

b.

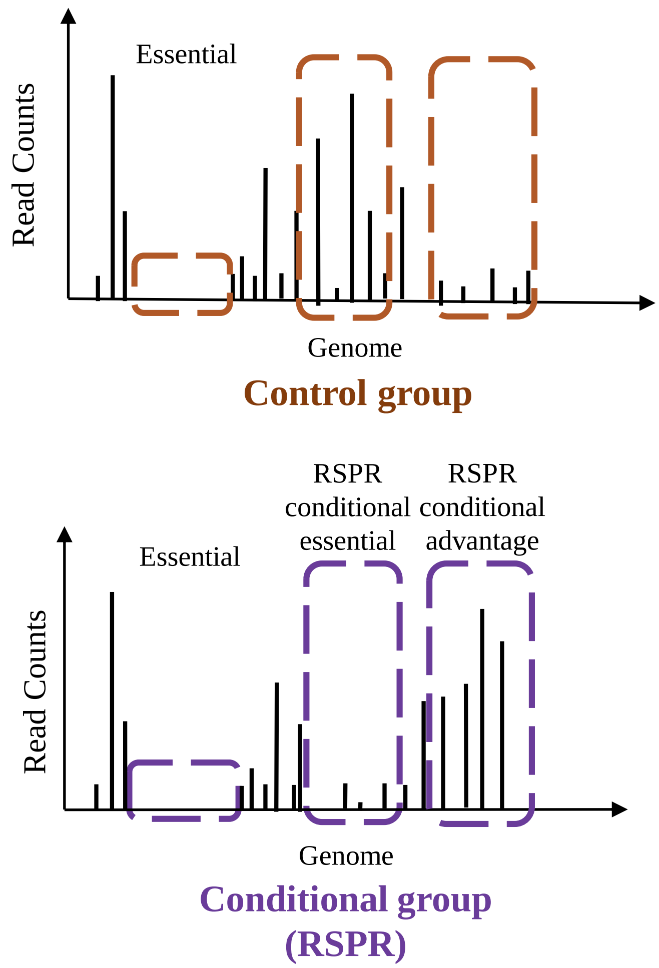

Figure S1 Cartoon illustrating conditional essentiality analysis by Tnseq. Essential genes of the input library (a) and compared with the selection condition (b) by mapping transposon insertion reads. Conditional essential genes are essential in (b) but not (a) and conditional advantage genes have enriched transposon insertions in (b) compared with (a).

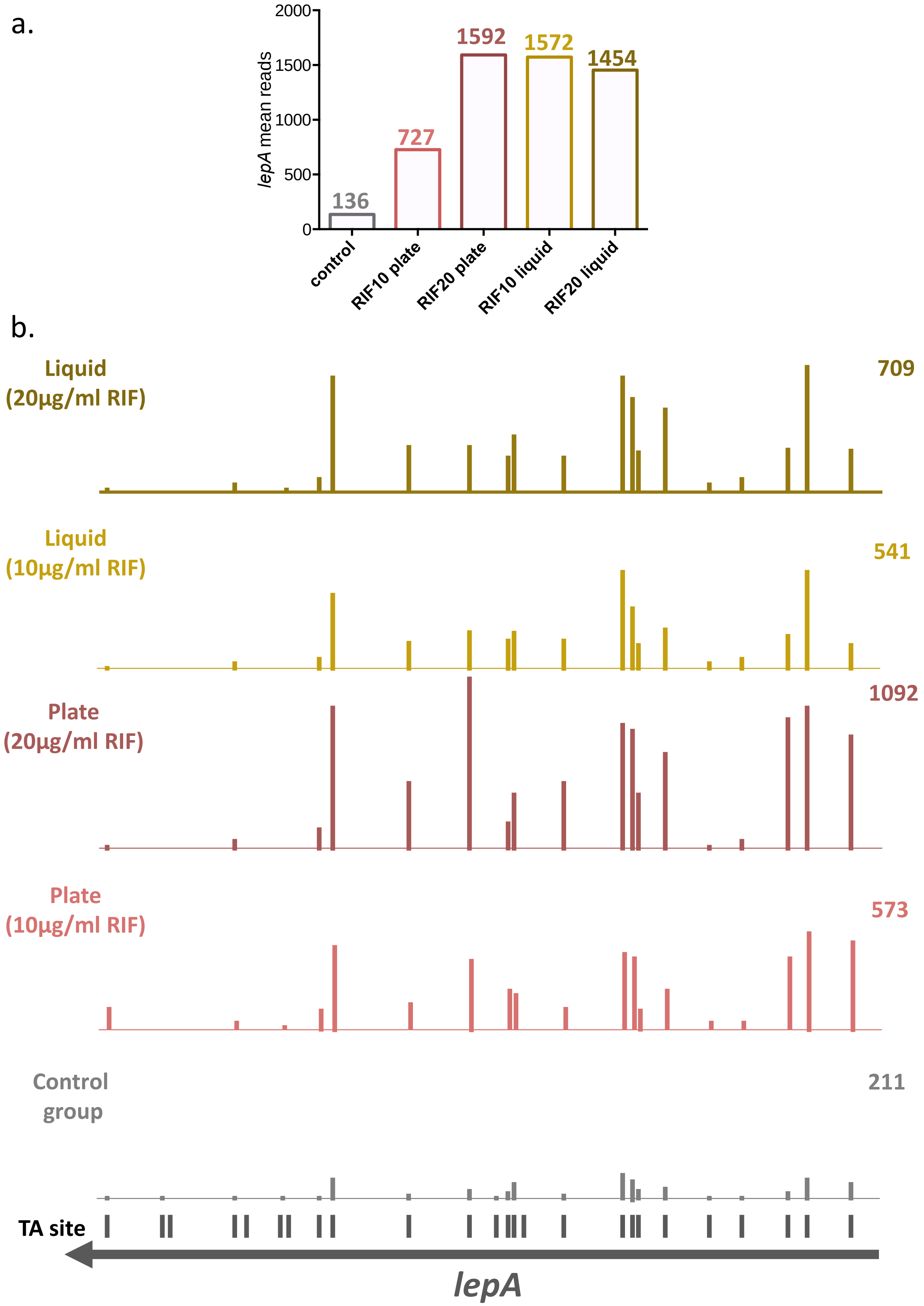

Figure S2. Transposon insertion reads in *lepA*. (a) Mean reads mapping to *lepA* by resampling using TRANSIT tool under the four selection conditions compared with the input library (control). (b) Relative read maps of the transposon insertions in *lepA* by TA site in the five conditions. The number to the right of the axis represents the maximum read count for any one TA locus.

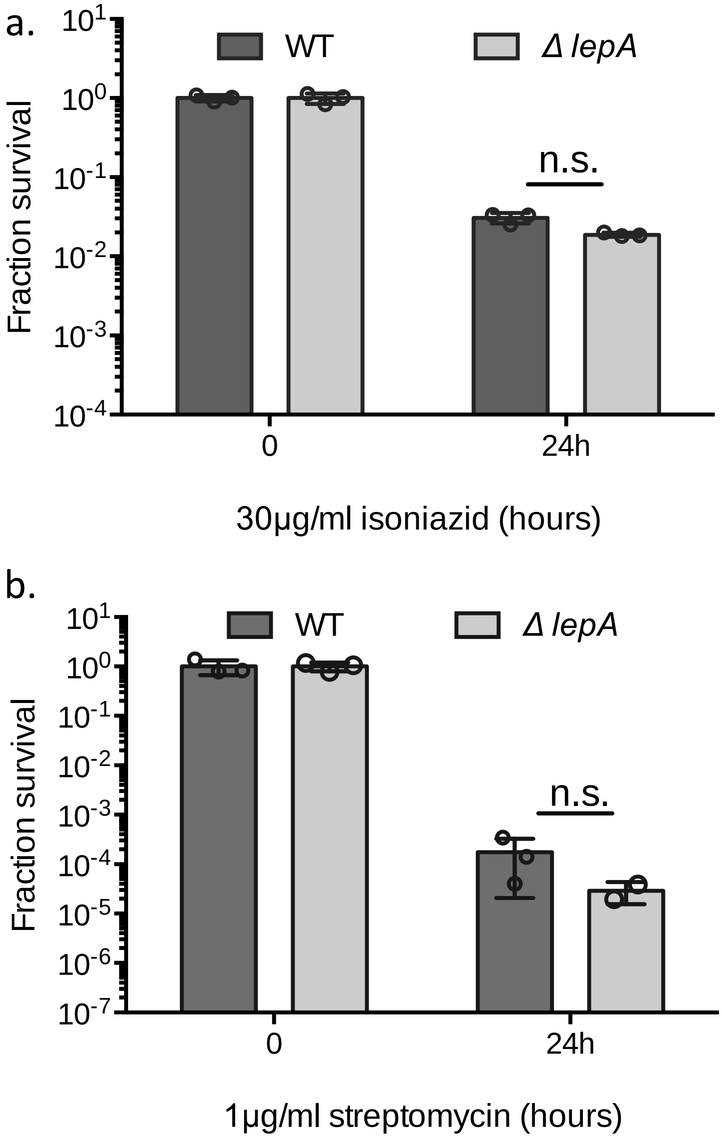

Figure S3. Deletion of LepA does not lead to generalized antibiotic tolerance. WT *M. smegmatis* and *M. smegmatis*-Δ*lepA* (Δ*lepA*) in axenic culture were treated with isoniazid (a) or streptomycin (b) at the indicated concentrations for 24 hours. Following treatment, bacteria were washed and plated on antibiotic-free agar and colonies counted after 4 days. Data are normalized to 1 for time=0. ns $p>0.05$ by Student's t-test.

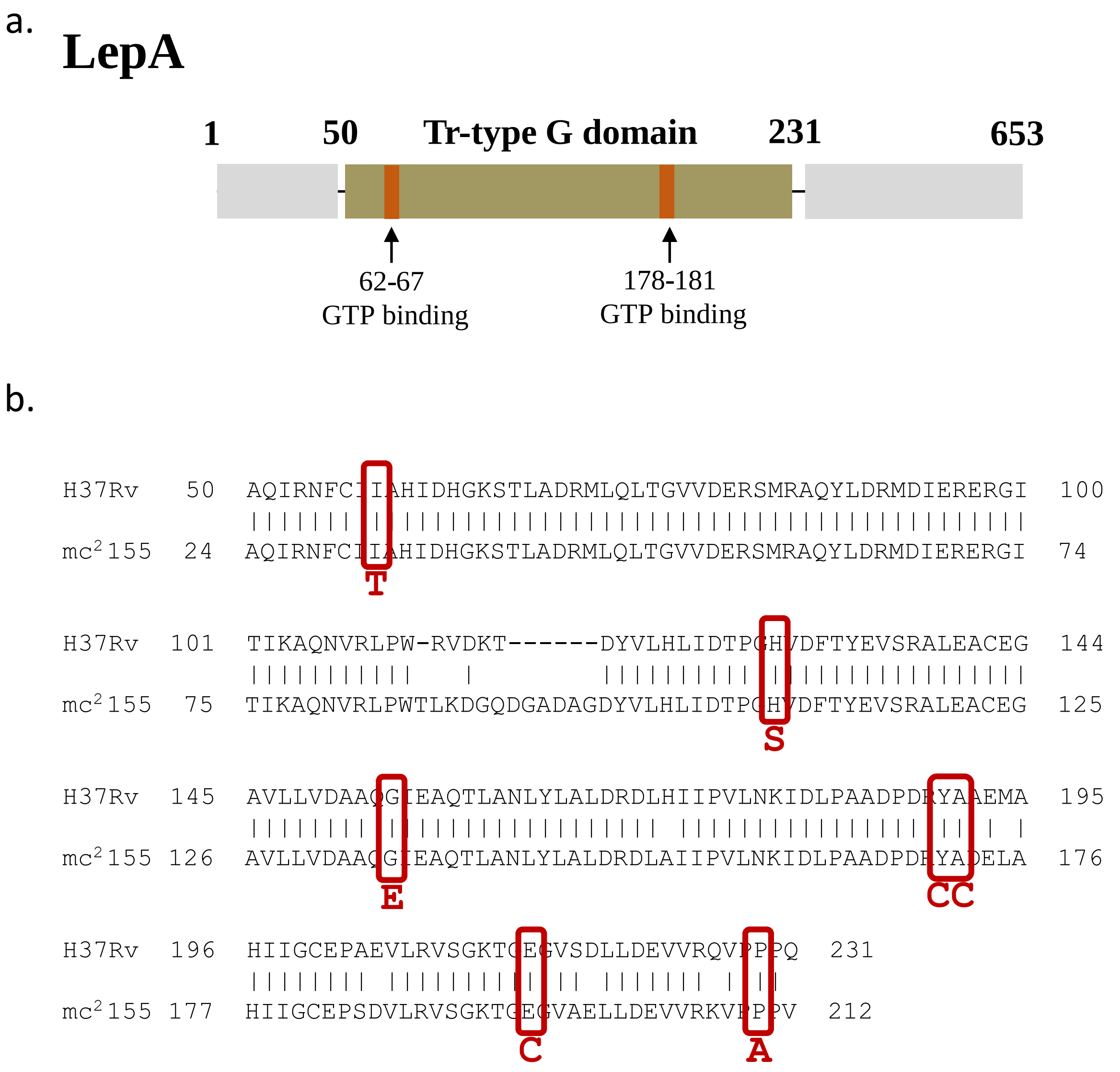

Figure S4. Mutations in conserved sites in *lepA* from clinical isolates used in RSPR assay. (a) Cartoon representing the LepA protein with the highly conserved GTP-binding domain shaded. (b) Amino acid alignment of part of the LepA sequence from *M. tuberculosis*-H37Rv and *M. smegmatis* mc^2-155. Mutations in *lepA* from clinical isolates corresponding to conserved amino-acids that were likely to disrupt function were chosen for testing and are illustrated in red (and see Dataset S1).

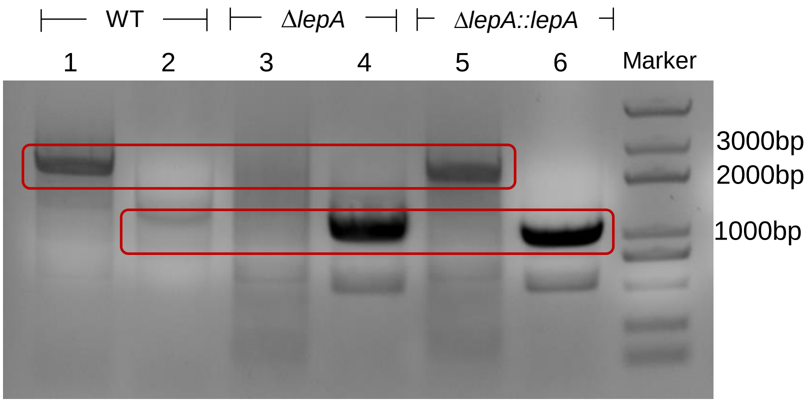

Figure S5. PCR validation of *lepA* deletion and complementation. 1% Agarose gel electrophoresis of DNA derived from WT, Δ*lepA* and Δ*lepA::lepA* as indicated. Lanes 1/3/5 represent amplification products of primer HindIII_lepA_F(500bp upstream of lepA ORF) and XbaI_lepA_R(the end of lepA ORF), representing the wild-type locus and lanes 2/4/6 represent amplification products of primer HindIII_lepA_F(500bp upstream of *lepA* ORF) and Zeo_R (the end of zeocin resistant gene) representing the recombineered deletion locus.
